# Fractionated Irradiation of Right Thorax Induces Abscopal Damage on Bone Marrow Cells via TNF-α and SAA

**DOI:** 10.3390/ijms22189964

**Published:** 2021-09-15

**Authors:** Yimeng Song, Songling Hu, Junling Zhang, Lin Zhu, Xinrui Zhao, Qianping Chen, Jianghong Zhang, Yang Bai, Yan Pan, Chunlin Shao

**Affiliations:** Institute of Radiation Medicine, Shanghai Medical College, Fudan University, Shanghai 200032, China; songyimeng@fudan.edu.cn (Y.S.); 18111140002@fudan.edu.cn (S.H.); 16111140003@fudan.edu.cn (J.Z.); 15111140001@fudan.edu.cn (L.Z.); 19111140003@fudan.edu.cn (X.Z.); 17111140001@fudan.edu.cn (Q.C.); zjh551268@fudan.edu.cn (J.Z.); yangbai@fudan.edu.cn (Y.B.)

**Keywords:** thoracic irradiation, abscopal effect, bone marrow damage, TNF-α, SAA, ROS

## Abstract

Radiation-induced abscopal effect (RIAE) outside of radiation field is becoming more attractive. However, the underlying mechanisms are still obscure. This work investigated the deleterious effect of thoracic irradiation (Th-IR) on distant bone marrow and associated signaling factors by irradiating the right thorax of mice with fractionated doses (8 Gy × 3). It was found that this localized Th-IR increased apoptosis of bone marrow cells and micronucleus formation of bone marrow polychromatic erythrocytes after irradiation. Tandem mass tagging (TMT) analysis and ELISA assay showed that the concentrations of TNF-α and serum amyloid A (SAA) in the mice were significantly increased after Th-IR. An immunohistochemistry assay revealed a robust increase in SAA expression in the liver rather than in the lungs after Th-IR. In vitro experiments demonstrated that TNF-α induced SAA expression in mouse hepatoma Hepa1–6 cells, and these two signaling factors induced DNA damage in bone marrow mesenchymal stem cells (BMSCs) by increasing reactive oxygen species (ROS). On the other hand, injection with TNF-α inhibitor before Th-IR reduced the secretion of SAA and attenuated the abscopal damage in bone marrow. ROS scavenger NAC could also mitigated Th-IR/SAA-induced bone marrow damage in mice. Our findings indicated that Th-IR triggered TNF-α release from lung, which further promoted SAA secretion from liver in a manner of cascade reaction. Consequently, these signaling factors resulted in induction of abscopal damage on bone marrow of mice.

## 1. Introduction

Radiotherapy is now one of the most important treatments for various malignancies, including lung cancer, liver cancer, nasopharyngeal carcinoma, and other tumors [1,2,3]. However, radiation can cause not only direct damage to the targeted tissue but also induce some unexpected effects on distal nonirradiated tissues [4], referred to as the abscopal effect. For example, when rats were cranially irradiated with 2 Gy X-rays, DNA damage and apoptotic cell death could be induced in the spleen cells [5]. Jan et al. confirmed that localized paternal cranial irradiation could cause a significant accumulation of unrepaired DNA lesions in sperm cells, leading to a profound epigenetic dysregulation in the unexposed progeny [6]. The incidence of abscopal effects is critically determined by signaling factors from irradiated cells to distant cells. These effects were shown to be mediated directly by gap junction intercellular communication (GJIC) and/or soluble cellular factors excreted from irradiated cells, such as reactive oxygen species (ROS), reactive nitrogen species (RNS), nitric oxide (NO), cytokines, extracellular DNA and exosomes [4,5]. 

The biological function of radiation-induced abscopal effect (RIAE) is a double-edged sword. For a positive perspective, tumor irradiation can activate the immune system, resulting in the shrinkage or even regression of unexposed distant tumors [7]. On the other hand, RIAE can cause normal tissue damage, such as DNA damage and chromosome instability [8,9], and even induce tumor formation [10]. Therefore, it is necessary to take into account of the harmful RIAE in distant organs and the involved roles of immune activation. 

Radiation could influence immune-modulatory effects by inducing T cell activation, antigen-presenting cell activation, natural killer cell activation, and other immune responses [11]. As part of the hematopoietic and immune system, bone marrow plays an important role in systemic immunity and hematopoiesis, which is always stimulated after irradiation. Lower doses of direct radiation (0.5–4 Gy) can cause bone marrow suppression [12]. In the process of immune activation induced by radiation, TNF-α, the most primitive and pro-inflammatory cytokine in inflammation, was approved to be an oncotoxic factor rather than to augment the anti-tumor immune responses [13]. In addition, plenty of in vivo studies have shown that inhibition of TNF-α can ameliorate radiation-induced intestinal inflammation, oral mucositis, and liver damage [14,15,16]. Although studies have shown that TNF-α contributes to bone marrow cell apoptosis in the total-body irradiated mice [17], there is no literature confirming the role of cytokines, especially TNF-α, in thoracic irradiation induced bone marrow damage.

Our previous study has reported that fractionated irradiation of right thorax could induce abscopal damage of the ultrastructure of blood-testis barrier and then dramatically decreased the sperm number and vitality in mice testes [18,19]. However, the potential abscopal effect caused by the irradiation of localized thorax on bone marrow has never been addressed in detail. This study demonstrated for the first time that the high-dose of partial thoracic irradiation could induce abscopal damage in bone marrow of mice, and the signaling factors of this RIAE were further investigated.

## 2. Results

### 2.1. Thoracic Irradiation Induces Tissue Injury in Bone Marrow Cells

**Figure 1:**
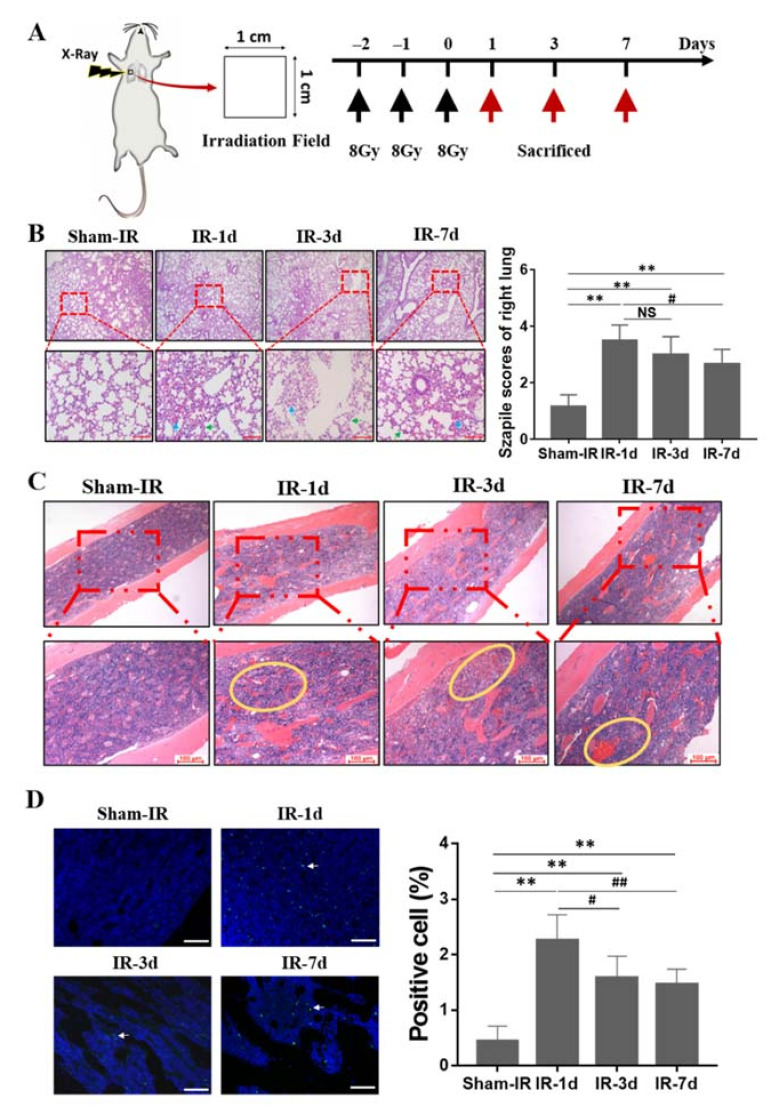
Thoracic irradiation (Th-IR) induces injuries in lung and bone marrow of mice. (**A**) The scheme of mouse irradiation and further analysis. 1 × 1 cm^2^ on the right chest of the mouse was irradiated with 8 Gy X-rays each day for three days. The lung and bone marrow cells of the mice were taken for further measurements at one, three, and seven days after irradiation. (**B**) HE staining and Szapiel scores for the pulmonary alveolitis and inflammation of the irradiated lung. Green arrows indicate thickened alveolar wall. Blue arrow indicates hemorrhage exudate. Bar = 100 μm. (**C**) HE staining of bone marrow tissue section. Yellow circles indicate tissue bleeding and damage. (**D**) TUNEL staining of bone marrow tissue section and the ratio of apoptotic cells in population. White arrows represent the TUNEL positively stained cells. Bar = 100 μm. ** *p* < 0.01, compared with sham-IR group. # *p* < 0.05 and ## *p* < 0.01 compared with IR-1d group. NS: non-significant. *n* = 8–10.

To study the abscopal effect of lung irradiation, an animal model was established by irradiating the right lung of mouse with fractionated doses of X-rays for three consecutive days (8 Gy/per day, three days), then the lung, femur and blood of the mice were collected at day one, three, and seven after this thoracic irradiation (Th-IR) for further analysis (Figure 1A). Figure 1B illustrates that, compared with the sham-IR group, Th-IR (8 Gy × 3) caused substantial damage in the irradiated lung tissue, including obvious inflammatory cell infiltration, alveolar structural damage and other pathological changes. Based on the Szapiel method, the severity of lung injury in the Th-IR group approached to 3–3.5 at day 1 post-irradiation. However, the inflammatory cell infiltrates, hemorrhage exudate, and thickened alveolar wall was progressively decreased along with the time after Th-IR. Then, we checked the changes of structural composition and histological architecture of mice bone marrow. As shown in Figure 1C, Th-IR induced severe hemorrhage in bone marrow, reduced the density of bone marrow cells, and loosed the structure of bone marrow (yellow circle). TUNEL assay showed that the apoptotic rate of bone marrow cells increased from 0.42 of control to 2.1% at day 1 after Th-IR and maintained at 1.58% and 1.42% at day three and day seven after Th-IR, respectively, which was still higher than that of sham-IR group (Figure 1D).

### 2.2. Th-IR Induces Bone Marrow Cell Damage and Cell Cycle Alteration

**Figure 2:**
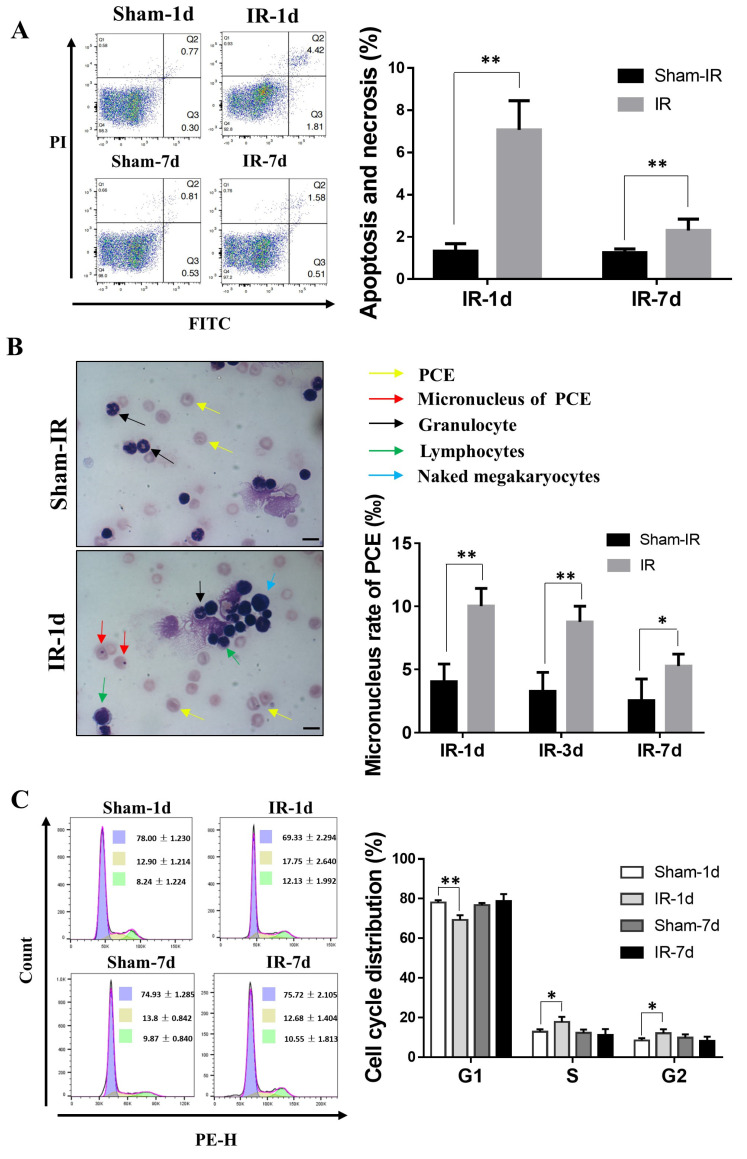
Th-IR induced cellular damage and cell cycle alteration in abscopal bone marrow of mice. (**A**) The bone marrow cell apoptosis and necrosis were enhanced after Th-IR. (**B**) Micronucleus frequency of PCEs was increased after Th-IR. Yellow arrow, PCEs; red arrow, micronucleus of PCEs (×1000). Bar = 10μm. (**C**) Th-IR decreased the percentage of the bone marrow cells in G1-phase and increased in S- and G2-phase at day 1 after Th-IR. * *p* < 0.05 and ** *p* < 0.01 compared with corresponding sham-IR group. *n*  =  8–10.

To further demonstrate the RIAE on bone marrow, we collected bone marrow cells and detected apoptosis induction, cell cycle distribution and micronucleus formation in polychromatic erythrocytes (PCEs) at different time points after Th-IR. As shown in Figure 2A, the apoptosis and necrosis rate of bone marrow cells increased from 1.01% of nonirradiated control to 8.46 % (*p* < 0.01) and 2.84% (*p* < 0.05) at day one and day seven after Th-IR, respectively. The micronucleus frequency of PCEs increased from 4% of nonirradiated control to 10% (*p* < 0.01) at day three after Th-IR and then declined to 5.25 % (*p* < 0.05) at day seven after Th-IR, suggesting that PCEs in mice suffered repairable DNA damage due to Th-IR (Figure 2B). Cell cycle analysis revealed that, after Th-IR, the bone marrow cell replication remained in the S- and G2-phase, and the cells in G1-phase decreased accordingly at day one after Th-IR (Figure 2C). These results indicated that Th-IR induced DNA damage, apoptosis, and the blockage of proliferation on the abscopal bone marrow cells in femur.

### 2.3. Proteomic Analysis of Differentially Expressed Proteins in the Serum of Mice after Th-IR

**Figure 3:**
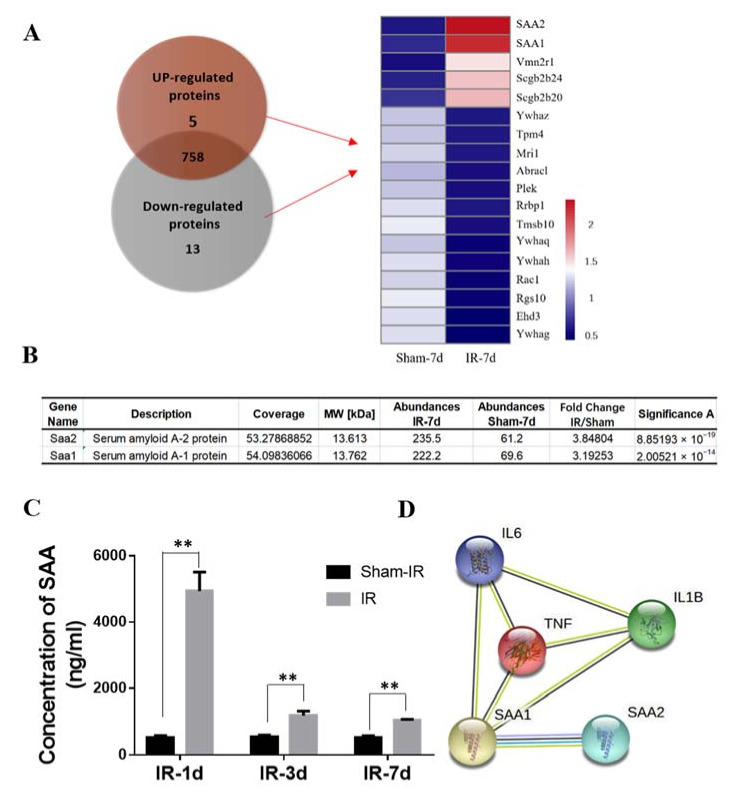

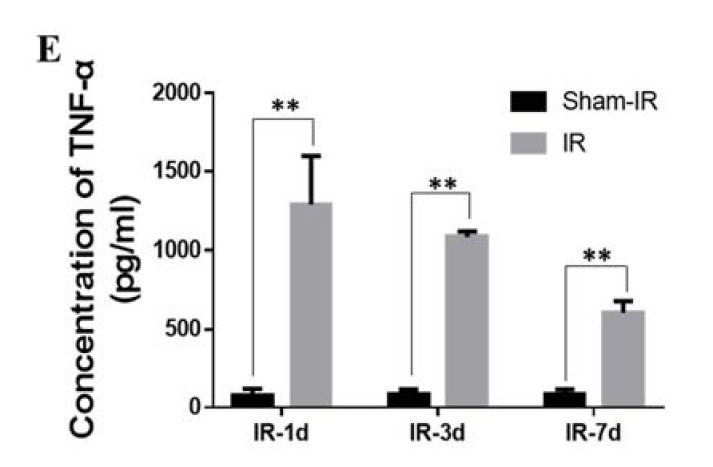
Changes of protein expressions in mouse serum after Th-IR. (**A**) Veen diagram and heat-map analysis of up-regulated proteins (>2 fold) and down-regulated proteins (<1/2 fold) between Th-IR and sham-IR group. (**B**) The expressions of SAA2 and SAA1 were up-regulated in the Th-IR group. (**C**) The concentration of SAA1 in mice serum at different time points after Th-IR. (**D**) STRING online analysis of protein-protein interaction (PPI) among SAA1, SAA2 and inflammatory factors. (**E**) The concentration of TNF-α in mice serum at different time points after Th-IR. ** *p* < 0.01, compared with sham-IR group. *n* = 6–8.

To know the potential signaling factors involved in the RIAE, tandem mass tags (TMT) technology was used to detect the proteomic profiles of mice serum of the sham-group and IR group at day seven after Th-IR. A total of 776 proteins were detected (Table S1) where the proteins with expression differences greater than 2-fold (up/down) are considered as differentially expressed proteins (DEPs). Among which, there were 18 DEPs (5 up-regulated and 13 down-regulated) between Th-IR and sham-IR group (Figure 3A). Notably, the expressions of serum amyloid A (SAA1 and SAA2) in the irradiated mice serum had the highest up-regulation folds (3.84 and 3.19, respectively) in comparison with sham-IR group (Figure 3B), indicating that these two factors may play vital roles in RIAE on bone marrow. Further measurement showed that the concentration of SAAs increased to 9-fold of nonirradiated control at day 1 after Th-IR and then decreased to 2-fold of control at day three and day seven after Th-IR (Figure 3C), indicating that the high inflammation level can be maintained in mice for a long time after Th-IR.

Both SAA1 and SAA2 are generally produced in hepatocytes, thus we assumed that the hepatocytes may be infected with signaling inflammatory factors released from the irradiated lungs and the produced SAAs were transported through blood circulation. To know the potential inflammatory factors involved in SAA induction, the possible cross interactions between SAAs and inflammatory factors were predicted by the online tool of String (version 11.0). It was found that TNF-α has a close relationship with SAA1 (Figure 3D). Then, we detected the concentration of TNF-α in the mice serum after Th-IR, and found that it is upregulated to 10-fold, 8-fold and 5-fold at day one, three and seven after Th-IR, respectively (Figure 3E), which had a similar time-response with the production of SAA in the mice serum.

### 2.4. Effect of Exogenous SAA on Bone Marrow Mesenchymal Stem Cells

We then wanted to know how SAA induces bone marrow damage. We isolated and purified the bone marrow mesenchymal stem cells (BMSCs) according to the protocol proposed by Huang et al. [20], and detected the ROS production and DNA damage in BMSCs after SAA treatment. Supplementary Figure S1A showed that BMSCs displayed a fibroblast-like, spindle-shaped morphology at the third generation, and oil-red-O, and Alizarin Red staining ascertained the differentiation capabilities of BMSCs to osteoblast and adipocytes. Moreover, the purified BMSCs presented positive biomarkers of CD44 and CD90 but negative CD31 and CD45 (Supplementary Figure S1B).

**Figure 4:**
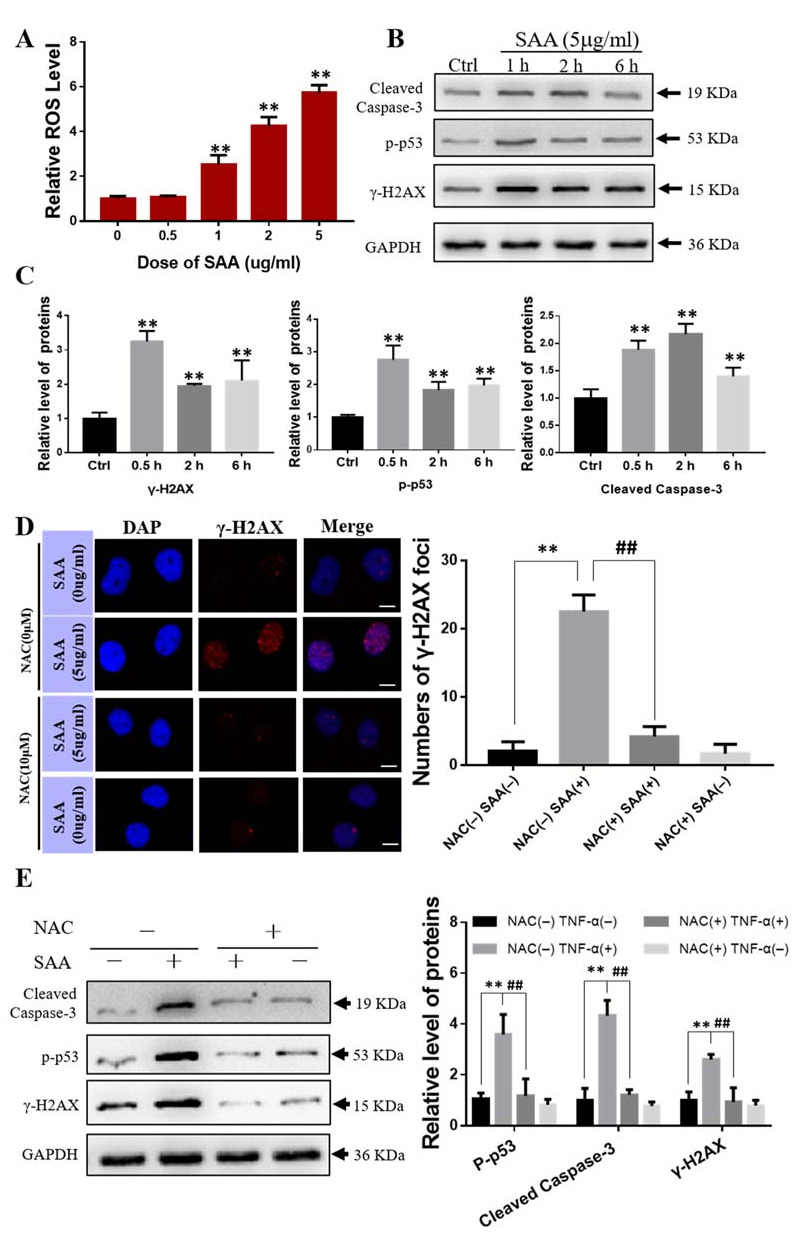
The effects of exogenous SAA on BMSCs. (**A**) Relative level of ROS in BMSCs treated with 0–5 μg/mL of SAA for 1 h. ** *p* < 0.01 compared with control group. (**B**,**C**) Western blot analysis of the protein expression of Cleaved caspase-3, p-p53 and γ-H2AX in BMSCs after the treatment with SAA. ** *p* < 0.01 compared with control group. (**D**) NAC mitigated SAA-induced -H2AX foci formation in BMSCs. Cells were treated with 10 μM NAC for 0.5 h before SAA treatment for 1 h. Bar = 10 μm. (**E**) Western blot assay of the protein expressions of Cleaved caspase-3, p-p53 and -H2AX in BMSCs treated with SAA and/or NAC. ** *p* < 0.01 compared with NAC (−) SAA (−) group; ## *p* < 0.01 compared with NAC (+) SAA (+) group. Each experiment was repeated at least three times.

It was found that when BMSCs were treated with exogenous SAA (0.5–5 g/mL) for 1 h, the level of intracellular ROS increased along with the concentration of SAA (Figure 4A). Western blot assay showed that the protein expressions of γ-H2AX, p-p53 and Cleaved Caspase-3 were significantly increased in the SAA treated BMSCs (Figure 4B,C). Meanwhile, the number of γ-H2AX foci in BMSCs was drastically increased after SAA (5 g/mL) treatment but reduced to a very low level when the cells were pretreated with a ROS scavenger, NAC (N-Acetyl-L-cysteine) (10 μM) for 0.5 h before SAA treatment for 1 h (Figure 4D). Western blot assay also demonstrated that the SAA treatment increased the activation of DNA damage and apoptosis-related proteins (γ-H2AX, p-p53 and cleaved-caspase3), however, the ROS scavenger NAC mitigated the over expressions of these proteins (Figure 4E). Accordingly, SAA induced cell damage by increasing the production of intracellular ROS.

### 2.5. Effect of TNF-α on Bone Marrow Mesenchymal Stem Cells

**Figure 5:**
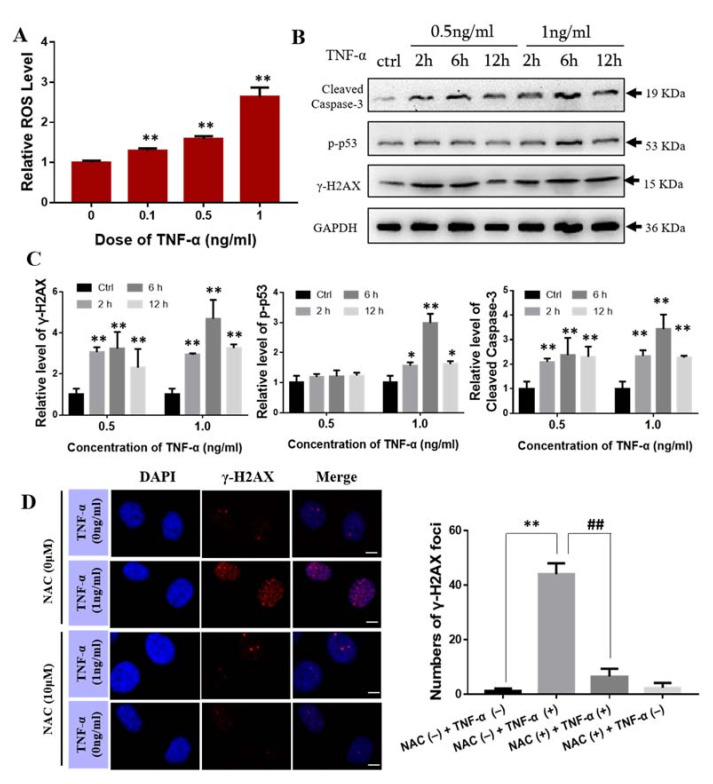

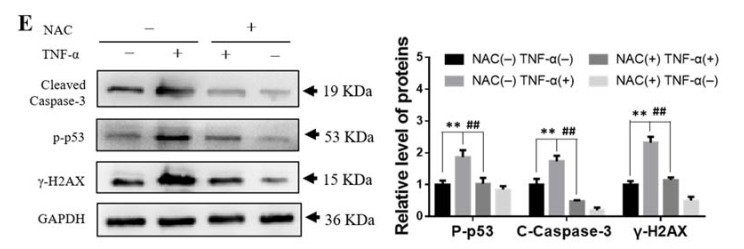
Scavenging ROS decreased DNA damage in BMSCs induced by TNF-α. (**A**) Relative ROS level in BMSCs treated with 0 or 1 ng/mL of TNF-α for 1 h. ** *p* < 0.01 compared with control group. (**B**,**C**) Western blot analysis of the expression of Cleaved caspase-3, p-p53 and γ-H2AX protein in BMSCs after the treatment with TNF-α. * *p* < 0.05 and ** *p* < 0.01 compared with control group. (**D**) Immunofluorescence staining of γ-H2AX in BMSCs in the presence and absence of NAC (10 μM) for 0.5 h before exposure of TNF-α (1 ng/mL) for 6 h. Bar = 10 μm. (**E**) Western blot analysis of the expression of p-p53, Cleaved caspase-3 and γ-H2AX protein in BMSCs treated with TNF-α and/or NAC. ** *p* < 0.01 compared with NAC (−) TNF-α (−) group; ## *p* < 0.01 compared with NAC (+) TNF-α (+) group. Each experiment was repeated at least three times.

Next, we examined the effect of exogenous TNF-α on BMSCs. When BMSCs were treated with TNF-α, the intracellular ROS level was increased with the concentration of TNF-α (Figure 5A), and the expressions of γ-H2AX, p-p53 and Cleaved Caspase-3 were also up-regulated in a manner of dose- and time- dependent (Figure 5B,C). To know the function of ROS in TNF-α induced DNA damage, BMSCs were pretreated with NAC (10 μM) for 0.5 h before TNF-α (1 ng/mL) treatment for 6 h. Expectedly, this ROS scavenger decreased the number of γ-H2AX foci in the TNF-α treated BMSCs (Figure 5D). Western blot assay also showed that inhibition of ROS production by NAC effectively attenuated TNF-α induced γ-H2AX expression and reduced the protein levels of p-p53 and cleaved caspase-3 in BMSCs (Figure 5E). Collectively, these results revealed that TNF-α could induce DNA damage in BMSCs via the activation of ROS production.

### 2.6. TNF-α Induces the Expression of SAA In Vivo and In Vitro

**Figure 6:**
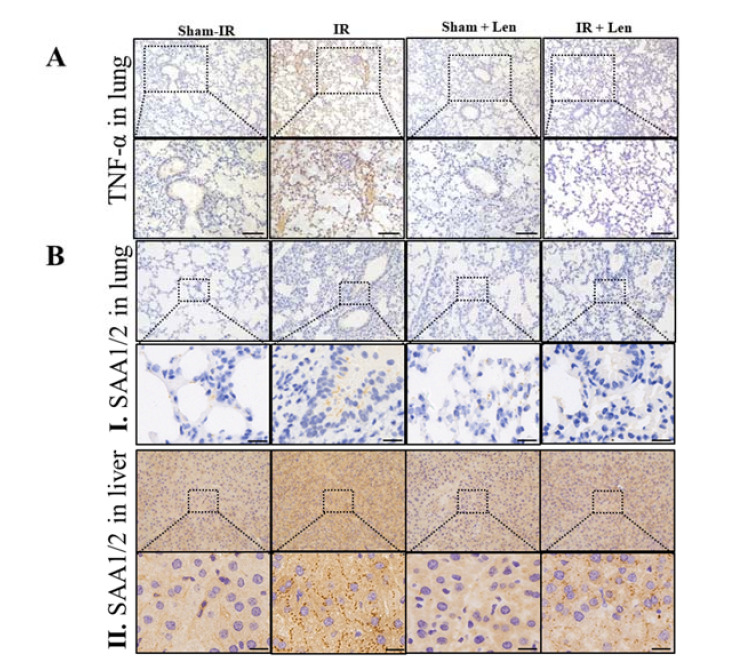

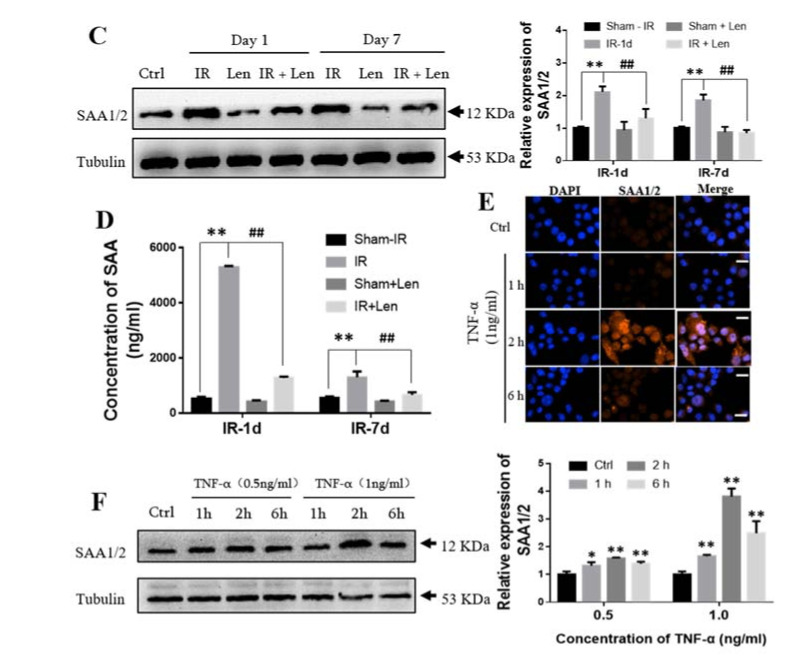
TNF-α induces the expression of SAA in vivo and in vitro. For in vivo experiments, mice were pretreated with or without TNF-α inhibitor lenalidomide (50 mg/kg/d, len) by intraperitoneal injection before Th-IR (8 Gy × 3). The serum and liver were collected at different time points after Th-IR. For in vitro experiments, the expressions of SAA1/2 were observed in mouse hepatoma cells Hepa1–6 treated with TNF-α. (**A**) Immunohistochemical staining of TNF-α in lung tissue at day one after Th-IR. Bar = 100 μm. (**B**) Immunohistochemical staining of SAA1/2 in lung and liver tissue section of mice with indicated treatment at day 1 after Th-IR. Bar = 25 μm. (**C**) Western blot analysis of the expression of SAA1/2 protein in the liver of mice with indicated treatment. (**D**) The concentration of SAA in the serum of mice with indicated treatment. ** *p* < 0.01 indicated a significant difference with sham group, ## *p* < 0.01 indicated a significant difference with the IR group. (**E**) Immunofluorescence staining of SAA in Hepa1–6 cells after the treatment with TNF-α. Bar = 25 μm. (**F**) Western blot analysis of the expression of SAA1/2 protein in Hepa1–6 cells after the treatment with TNF-α. * *p* < 0.05 and ** *p* < 0.01 compared with control group. *n* = 8–10 for mice experiments. All in vitro experiments were repeated at least three times.

Both TNF-α and SAA could induce DNA damage in BMSCs by inducing ROS, then we explored the relationship between TNF-α and SAA in vivo. For this purpose, we injected intraperitoneally TNF-α inhibitor of lenalidomide (Len) into mice before Th-IR. Compared with mice that received only Th-IR, Len injection extensively decreased the expression of TNF-α in the lung tissue at day one after Th-IR (Figure 6A). Further analysis revealed that TNF-α concentration was decreased by 81%, 80% and 56% in the serum of Len-injected mice in comparison with those in the irradiation group at day one, three, and seven after Th-IR, respectively (Supplementary Figure S2).

As acute stress-responsive proteins, SAA1/2 are mainly expressed in liver during the acute phase response (APR). To validate this conjecture, we examined the expression of SAA1/2 in the lung and liver tissues after irradiation and/or injection of Len. Figure 6B illustrates that the expression level of SAA1/2 in the lung tissues of all groups was extremely low. However, the immunohistochemistry and Western blot assay demonstrated that the expression of SAA1/2 in hepatocytes was significantly increased after Th-IR, and the treatment of Len significantly reduced SAA1/2 in the liver of irradiated mice (Figure 6B,C). As shown in Figure 6D, in comparison with Th-IR alone group, the concentration of SAA in the liver of Len-injected mice decreased by 78% and 48% at one and seven days after irradiation, respectively. Further in vitro immunofluorescence and Western blot assays revealed that TNF-α could induce SAA1/2 expression in mouse hepatoma cells Hepa1–6 (Figure 6E,F). These results indicate that SAA1/2 is mainly produced by liver cells after interacting with peripheral inflammatory TNF-α released from irradiated lung of mice.

### 2.7. TNF-α Contributes to the Abscopal Bone Marrow Damage in the Th-IR Mice

**Figure 7:**
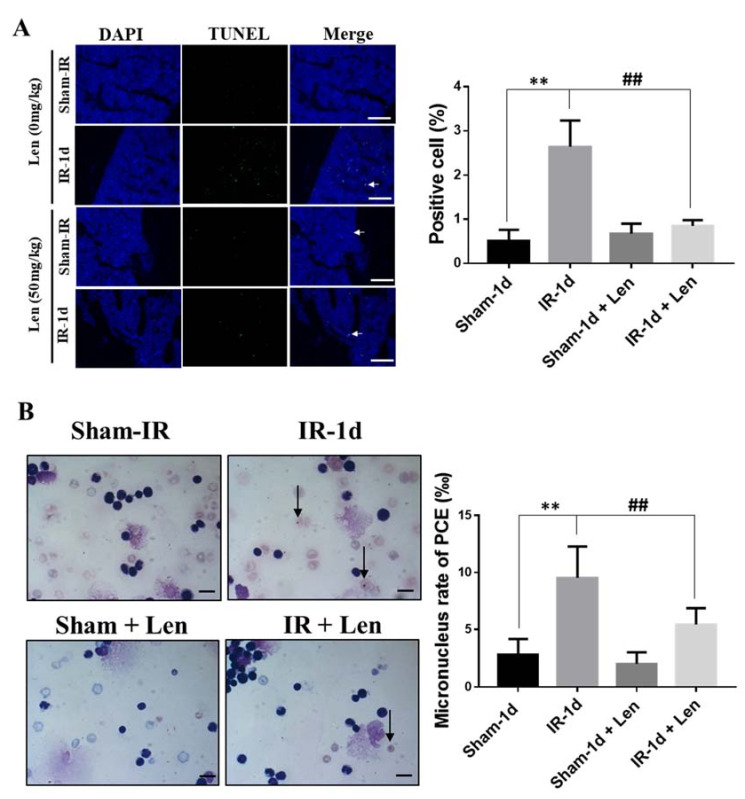
TNF-α contributes to the abscopal bone marrow damage in the Th-IR mice. Mice were pretreated with or without TNF-α inhibitor of lenalidomide (50 mg/kg, Len) by intraperitoneally injection before Th-IR (8 Gy × 3). The bone marrow of mice was collected at day 1 after Th-IR. (**A**) TUNEL staining and apoptosis rate of bone marrow cells. White arrows represent the TUNEL positively stained cells. Bar = 100 μm. (**B**) PCEs staining and micronucleus formation. Black arrow, micronucleus of PCEs. ** *p* < 0.01 indicated a significant difference from sham group; ## *p* < 0.01 indicated a significant difference with the IR group. Bar = 10μm. *n* = 8–10.

We further verified that TNF-α participated in the RIBE on bone marrow. An intensive burst of cellular apoptosis was observed in mice bone marrow at day one after Th-IR, but it was significantly reduced in the Len-treated mice (Figure 7A). Meanwhile, the micronucleus formation in bone marrow PCEs of irradiated mice was also reduced by lenalidomide (Figure 7B). These results confirmed that TNF-α contribute to Th-IR induced damage in abscopal bone marrow.

### 2.8. ROS Scavenger NAC Eliminated Th-IR/SAA-Induced Bone Marrow Damage in Mice

**Figure 8:**
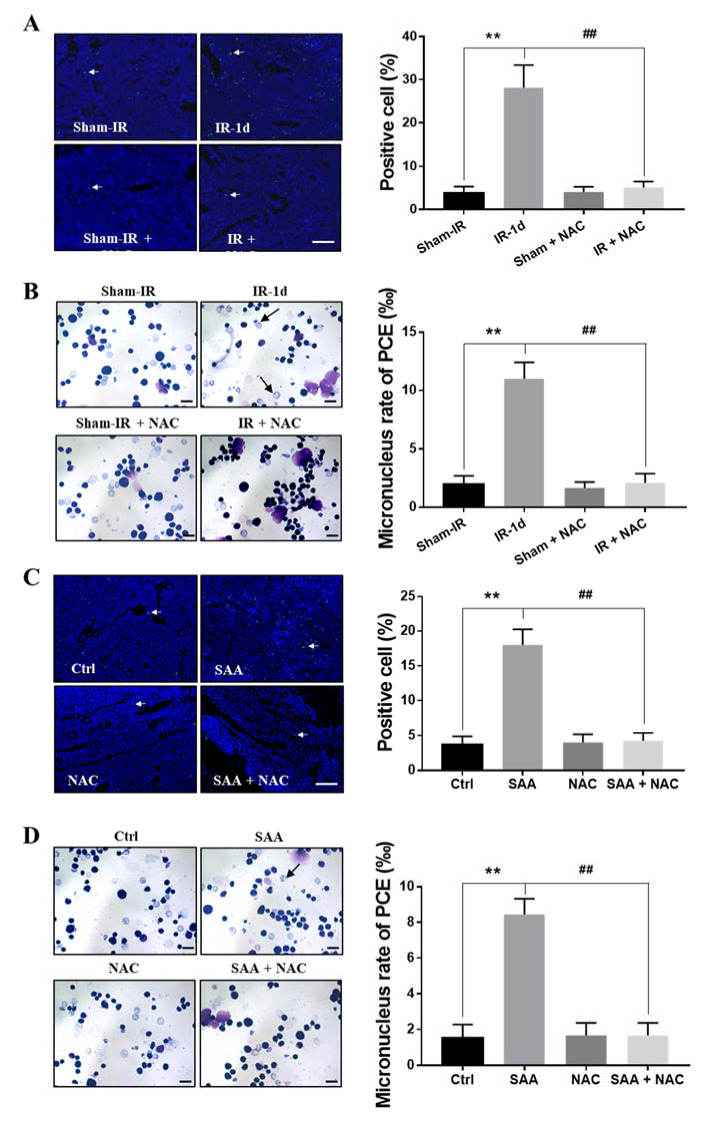
ROS scavenger NAC eliminated Th-IR/SAA-induced bone marrow damage in mice. The mice were injected subcutaneously daily with NAC (100 mg/kg, dissolved in PBS) for three days before Th-IR (8 Gy × 3) or SAA treatment (intraperitoneally injection, 0.35 mg/kg). The bone marrow of mice was collected at day one after Th-IR or SAA injection. (**A**,**C**) TUNEL staining and TUNEL-positive cells. White arrows represent the TUNEL positively stained cells. Bar = 100 μm. (**B**,**D**) PCEs staining and micronucleus formation. Black arrow, micronucleus in PCEs. ** *p* < 0.01 indicated a significant difference with sham/Ctrl group; ## *p* < 0.01 indicated a significant difference with IR/SAA group. Bar = 10μm. *n* = 8–10.

Irradiation and SAA induce substantial production of ROS. Next, we further investigated whether inhibition of ROS with N-acetyl-L-cysteine (NAC) could block the damage effects of Th-IR/SAA on bone marrow cells. After one day of Th-IR, the percentage of TUNEL-positive cells was increased in bone marrow of irradiated mice, but this increase was eliminated by NAC injection (Figure 8A). Meanwhile, PCEs staining revealed comparable result that Th-IR induced micronucleus formation in PCEs was significantly attenuated by treatment with NAC (Figure 8B). On the other hand, when the mice were treated with SAA, cellular apoptosis and micronucleus could be induced in bone marrow and PCEs, respectively, but these damage effects were effectively eliminated by NAC treatment (Figure 8C,D). Accordingly, both Th-IR and SAA treatment resulted in cell apoptosis and DNA damage in bone marrow cells by inducing ROS generation in mice.

## 3. Discussion

RIAE is a double-edged sword to radiation health. A large number of studies have shown that irradiation of primary tumors could induce the shrinkage or even regression of distant tumors, suggesting that RIAE is beneficial to cancer patients [7]. However, some other studies have confirmed that RIAE could induce distal normal tissue damage [8,21] and even induce tumors [10], implying that RIAE is harmful. In this study, we found that the fractionated irradiation on the right thorax could induce abscopal effect on distal femur bone marrow, including changes of bone marrow structure, induction of apoptosis and DNA damage, which indicates an extension of the detrimental effects of Th-IR. To our knowledge, this study demonstrated for the first time that Th-IR could induce bone marrow damage in mice. Moreover, we disclosed that the distal bone marrow cell damage resulted from the upregulation of irradiation induced inflammatory factor of lung TNF-α and its downstream cascade factor of liver SAA.

Bone marrow cells are exquisitely sensitive to irradiation, and 2.4–4 Gy radiation can lead to noticeable DNA damage and apoptosis in bone marrow cells [22,23]. It was reported that local irradiation of mice [24], injecting exosomes collected from irradiated cells into mice through tail vein [25], or transplanting irradiated bone marrow cells into mice [26,27] can cause DNA damage or chromosomal instability in unirradiated bone marrow cells. The current study found that the Th-IR could induce DNA damage and apoptosis in bone marrow cells within seven days after Th-IR. These damages were triggered immediately at day 1 after Th-IR and then gradually recovered at seven days after Th-IR, which is similar to direct radiation effect that the bone marrow cells can finish self-heal and renewal in a short period (72 h) after irradiation due to the ability of adaptive response stimulation [28].

SAA is a major acute-phase protein, which is mainly produced by hepatocytes during APR and then released into blood as a protein subtype of SAA1/2 [29]. Previous studies have confirmed that SAA is widely expressed in many extrahepatic tissues such as breast, stomach, small and large intestine, prostate, and lung, and its expression was localized predominantly to the epithelial components of a variety of tissues [30]. Nonetheless, as with other acute-phase reactants, the liver was proven to be the major site of SAA expression [31]. In stimulated mice, up to 2.5% of the synthetic capacity of liver may be directed to SAA protein synthesis [32]. Similarly, our study found that trace amounts of SAA expression was detected in local lung tissues, which was much lower than that in liver tissue of the irradiated mice.

SAA may play an indicating role for radiation. It was proposed that the dynamic change of the concentration of SAA might function as a prognostic indicator of the time course and severity of acute radiation sickness [33]. In this study, the proteomic analysis and ELISA assay demonstrated that the concentration of SAA in the mice serum was significantly increased after Th-IR. Similarly, it was reported that the level of SAA in mice serum approached to a peak level at day one after 2–8 Gy of total body irradiation and then gradually decreased to normal level within three days [34,35]. However, our study showed that the SAA concentration in the serum increased to the maximal level at day one but was still significantly higher than the control group at day seven after Th-IR, which indicated that a high dose of fractional irradiation could maintain inflammatory response in a relative long period.

SAA can lead to ROS generation in cells and further affect redox balance, activate caspases and DNase, and finally induce apoptosis [36]. This is in agreement with our study that exogenous SAA administration increased the intracellular ROS, activated the expressions of DNA damage related proteins, and induced apoptosis of BMSCs. While these SAA responses were alleviated by antioxidant of NAC. Similarly, our in vivo studies also proved that DNA damage induced by SAA in bone marrow cells was blocked by NAC.

In addition, keeping consistent with our previous studies [18,19], the key function of serum TNF-α in Th-IR induced abscopal effects was also verified in this work. Here, the cytokine assay demonstrated that, after Th-IR, the expression of TNF-α was significantly increased in the lungs and serum of mice. As a pro-inflammatory cytokine, TNF-α can be induced by irradiation and contributes to cellular apoptosis and necrosis induction, and regulates the bone marrow microenvironment in the irradiated mice [17,37,38]. TNF-α can enhance ROS accumulation and trigger caspase-8 and caspase-3 activation, which eventually leads to apoptosis [39,40,41]. Consistently, our data showed that TNF-α can induce ROS and cause DNA damage in BMSCs, while ROS scavenger protects against TNF-α induced apoptosis. Moreover, injection of TNF-α inhibitor into mice before Th-IR mitigated the damage in bone marrow cells, which proves that TNF-α may be a key signaling factor involved in the RIAE.

It is interesting to know the relationship between radiation-induced TNF-α and SAA. As shown in mass spectrometry data, the expressions of SAA in the irradiated mice serum had the highest up-regulation folds in comparison with sham-IR group. However, for other significantly up-regulated proteins, such as Vmn2r1 and Scgb2b24, to the best of our knowledge, no remarkable relationships with SAA have been found yet, and these proteins may not play important role in DNA damage or inflammatory response. Unlike other up-regulated proteins, SAA has been shown to be widespread in inflammatory events such as infection, trauma and neoplasia. SAA can be induced by inflammatory factors and regulate the inflammatory response via its receptors, predominantly TLR2/4 and FPR2, respectively [42]. We found that the induction of TNF-α and SAA in mice serum had a very similar dynamic time-response after Th-IR, suggesting a close relationship of them. In vitro experiments demonstrated that TNF-α could significantly increase the expression of SAA in Hepa1–6 cells, which is in consistent with the report that the production of SAA could be stimulated by TNF-α in HepG2 cells [43]. In addition, the TNF-α inhibitor reduced (albeit not completely) the expression of SAA in liver cells and decreased the concentration of SAA in mice serum after HF-IR, thus TNF-α may not the only factor involved in the production of SAA. Many cytokines such as TNF-α and IL-6 can induce SAA expression [29]. Radiation can induce TNF-α, as well as other cytokines that may promote expression of SAA. However, it has been demonstrated that TNF-α is the earliest produced inflammation factor that could further promote the expressions of other cytokines [44,45,46]. Therefore, our findings of the cascade reaction of cytokines and SAA may provide a new idea to solve the problem of radiation induced chronic inflammation in the later effect of irradiation. Clinical assessment and intervention for patients should encompass both factors, and anti-TNF/SAA treatment may reduce deleterious effects during radiotherapy.

**Figure 9:**
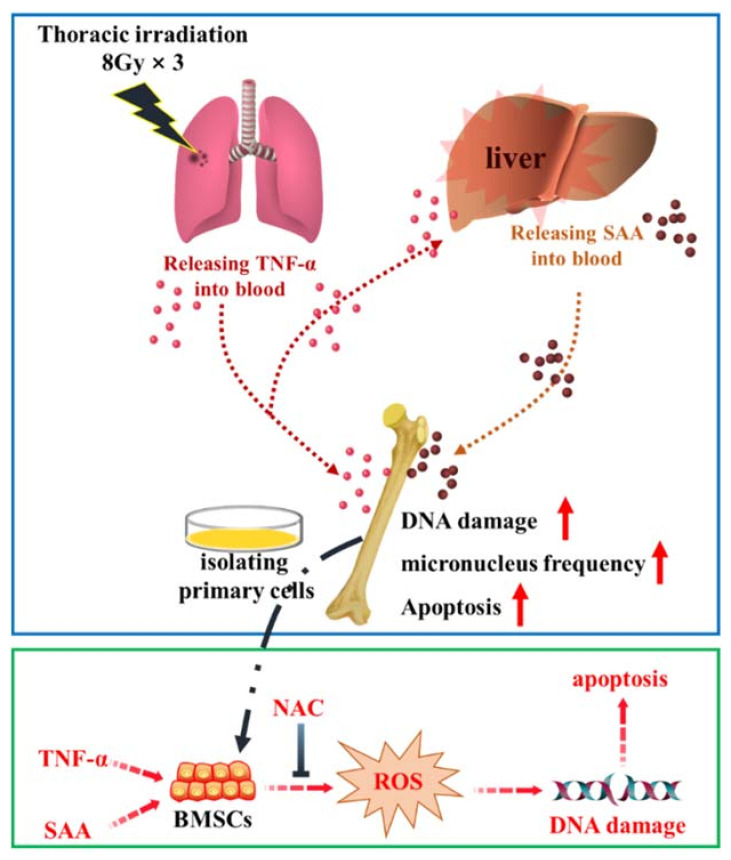
Schematic mechanism of fractionated irradiation of right thorax induced abscopal effects on bone marrow of mice. Irradiation on the right chest triggers the production of TNF-α which transports to liver via blood circulation and up-regulates SAA level in hepatocytes. Once transported to bone marrow, both TNF-α and SAA contribute to DNA damage and apoptosis of BMSCs via ROS formation.

In summary, Th-IR enhanced SAA expression in liver and induced abscopal damages in the distal bone marrow, and the inflammatory factors TNF-α and SAA might play key roles in these abscopal damages (Figure 9). During the development of the Th-IR induced abscopal responses, TNF-α was secreted by the irradiated lung cells and transported to liver via blood circulation, and further up-regulated SAA level in hepatocytes. Once transported to the bone marrow, both TNF-α and its downstream SAA contributed to DNA damage in bone marrow cells via ROS formation. Reduction of TNF-α secretion could attenuate the abscopal damage induced by Th-IR. Therefore, it is necessary to consider the management of inflammatory factors to eliminate the adverse effects of RIAE for better prognosis of radiotherapy regime.

## 4. Materials and Methods

### 4.1. Animal and Treatment

Six-week-old male C57BL/6 J mice were purchased from Shanghai SLAC Laboratory Animal Co. Ltd. (SLAC, Shanghai, China). All mice were housed with 12 h light-dark cycling. Mice were randomly divided into sham-irradiation group (sham-IR) and irradiation group with 8–10 mice each group. For the irradiation group, the right thorax of the mice were locally exposed to 8 Gy X-rays with a dose rate of 0.883 Gy/min (X-RAD 320, PXI Inc., North Branford, CT, USA) through a collimator (1 × 1 cm^2^) for three consecutive days (24 Gy in total). The mice from sham-IR group were covered with a lead thick enough to shield X-rays. In some experiments, mice were intraperitoneally injected with a TNF-α specific inhibitor, lenalidomide (50 mg/kg, Len) (CC-5013, Selleck, Houston, TX, USA) or N-acetyl-L-cysteine (NAC; Sigma, Darmstadt, Germany) (100 mg/kg, dissolved in PBS) before irradiation. All animal experiments were approved by the Animal Ethics Committee of Fudan University.

### 4.2. Detection of Bone Marrow Cell Apoptosis

At 1- and 7-days post-irradiation, mice were sacrificed and the femurs were quickly collected. Both ends of the femur were cut to blow the bone marrow into an EP tube with a syringe. The bone marrow solution was slightly blown to scatter the tissue into a single cell suspension. The cells were washed with PBS triply and stained with Annexin V-FITC/PI using the dead cell apoptosis kit (Becton, Dickinson and Company, Franklin Lakes, NJ, USA). Then cells were sorted with a flow cytometry (Beckman Coulter, Abrea, CA, USA) and analyzed with FlowJo software (version 10).

### 4.3. Cell Cycle Analysis of Bone Marrow Cells

Bone marrow cells were collected and fixed in 70% ethanol and stored at −20 °C for 24 h, then cells were washed with PBS and stained with a cell cycle reagent (BD, USA), and the cell cycle distribution was detected by a flow cytometry (CytoFLEX, Beckman Coulter, CA, USA) and analyzed with Flowjo software.

### 4.4. Measurement of Micronucleus in Polychromatic Erythrocytes (PCEs)

The bone marrow cells were resuspended in fetal bovine serum, dropt onto glass slide for smearing, dried naturally, fixed with a fixative solution (methanol: glacial acetic acid = 85:15) for 30 min, then stained with Giemsa solution. The micronucleus (dark purple) in 2000 PCEs (light purple) of each sample were counted using a microscopy under a 100× oil objective, and the percentage of PCEs with micronucleus was then calculated.

### 4.5. Bone Marrow Stem Cells (BMSCs) Isolation, Culture and Identification

Bone marrow cells were isolated from the femur of mice and cultured at at 37 °C in 5% CO_2_ circumstance for three passages, then some cells were cultured in adipogenic induction medium for 4 weeks and then stained with Oil-red-O to observe lipid-rich vacuoles. Some cells were cultured for 2 weeks in osteogenic differentiation induction medium and stained with Alizarin Red to observe mineralized nodules. Moreover, the expressions of CD44, CD90, CD31 and CD45 on BMSCs were detected by flow cytometry assay. All antibodies for above CDs were bought from Biolegend (San Diego, CA, USA).

### 4.6. Western Blot Assay

The proteins were subjected to electrophoretic separation with 10% or 12% SDS–PAGE and transferred onto PVDF membranes. The membrane was incubated in 5% skimmed milk powder and then incubated with the primary antibody overnight at 4 °C. The following antibodies were used in the experiments: phospho-H2AX (Ser139) antibody (1:1000, #80312, CST, USA), cleaved-Caspase-3 Antibody (1:1000, #9661s, Cell Signaling Technology, Boston, USA), Phospho-p53 (Ser15) (1:1000, #82530, CST, USA), and mouse serum amyloid A1/2 antibody (1:500, AF2948, R&D Systems, Minneapolis, MN, USA). After incubation with HRP-conjugated secondary antibodies (1:5000, Beyotime Biotechnology, Shanghai, China), all blots were detected with an ECL kit (Bio-Rad, Hercules, CA, USA), and the band images were analyzed with Quantity one software (BIO-RAD).

### 4.7. Histological Analysis

Femurs, lung and liver tissues were dehydrated to paraffin embedding and then sliced into 3–5 μm sections. After deparaffinized, the sections were stained with hematoxylin and eosin (H&E) and observed under an optical microscope. In particular, with the lung tissue images magnified for 200 times, the sections were blindly scored on a scale of 1–4 for assessing the severity of alveolar inflammation: (1) no alveolitis and little to no cellular infiltrates; (2) mild alveolitis, local monocyte infiltration in less than 20% of the whole lung, no obvious damage to alveolar structure; (3) moderate alveolitis, the area accounts for about 20% to 50% of the whole lung; (4) severe alveolitis, ≥50% area of the whole lung, with occasional consolidation of monocytes and/or bleeding in the alveolar cavity.

### 4.8. TUNEL Assay

The paraffin-embedded femur was sectioned and stained in situ with TUNEL kit (Roche, Basel, Switzerland). The cell images of the tissue were randomly captured by a fluorescence microscope (DFC450-C Leica, Wetzlar, Germany). About 10 visual fields were randomly selected and the apoptosis rate of the cells was calculated.

### 4.9. Tandem Mass Tagging (TMT) Proteomics Analysis

The serum protein samples were collected from the mice in sham-IR and Th-IR group at the seventh day after irradiation. Immediately afterwards, the samples were taken to remove high-abundance proteins and obtain low-abundance component solutions by using the corresponding species of serum-depleted high-abundance affinity column Mouse 3. Subsequently, SDT lysis solution was added and the mixture was incubated in a boiling water bath for 10 min and then centrifuged at 14,000× *g* for 15 min. The resulting proteins were prepared for LC-MS/MS measurement performed by Genechem Co. Ltd. (Shanghai, China). In brief, the proteins were loaded by the autosampler to the analytical column (Acclaim PepMap RSLC 50 μm × 15 cm, nano viper, P/N164943) (Thermo Fisher Scientific, Waltham, MA, USA) for separation and then were analyzed by Q Exactive plus mass spectrometer (Thermo Fisher Scientific). The Uniprot database (http://www.uniprot.org, accessed on 28 March 2019) was applied for the qualitative analysis of the mass spectrometry data, and the software Mascot2.6 and Proteome Discoverer2.1 were used for library identification and quantitative analysis.

### 4.10. Detection of Inflammatory Factors

The serum was isolated from whole blood using serum collection tubes and stored at –80 °C until use. TNF-α and SAA in the serum were measured using corresponding ELISA kit purchased from Lianke Biotech Co., Ltd. (Hangzhou, China) according to the manufacturer’s instructions.

### 4.11. ROS Measurement

The intracellular ROS level was measured using an ROS detection kit (S0033S, Shanghai Beyotime Biotechnology Co., Ltd., Shanghai, China). In brief, the adherent cells were treated with 10 μM 2,7-dichlorohydrofluorescin diacetate (DCFH-DA) at 37 °C for 30 min. After washing with PBS triply, the intensity of fluorescence in cells was detected at excitation/emission wavelengths of 488/525 nm.

### 4.12. Cell Immunofluorescence Assay

The cells were fixed in 4% paraformaldehyde for 30 min and permeabilized with immunofluorescence permeabilization solution for 15 min. Then the non-specific sites were blocked with 2% BSA for 1 h at room temperature and the cells were incubated with the primary phospho-H2AX (Ser139) antibody (1:100, #80312, CST, USA) at 4 °C overnight. Subsequently, cells were incubated with fluorescent secondary antibody anti-IgG rabbit Alexa Alexa Fluor^®^555 (1:600, Thermo Fisher Scientific) in the dark at room temperature for 1 h, and then the cell nuclei were incubated with DAPI dye solution (5 µg/mL) for 10 min. The foci were observed and recorded under a fluorescent microscope (Olympus, Tokyo, Japan).

### 4.13. Statistical Analysis

The animals were randomly assigned to different groups for all experiments. All data from at least three independent experiments were presented as means ± S.E.M. Significant differences between indicated groups were determined using student’s *t*-test. Bar charts was plotted by PRISM 7 (GraphPad, Bethesda, MD, USA). Hotmap was drawn in RStudio (v1.1.456. RStudio, Inc., Boston, MA, USA) with the ggplot2 packages. *p* < 0.05 was considered statistically significant.

## Data Availability

The raw data supporting the conclusions of this article will be made available by the authors, without undue reservation, to any qualified researcher.

## Supplementary Materials

The following are available online at https://www.mdpi.com/article/10.3390/ijms22189964/s1.
