# Supplementary material for: Fractionated Irradiation of Right Thorax Induces Abscopal Damage on Bone Marrow Cells via TNF-α and SAA
Source: Int J Mol Sci. 2021 Sep 15;22(18):9964. doi: 10.3390/ijms22189964 (PMC8468747; doi:10.3390/ijms22189964)
Supplement: Supplementary file 1 [file ijms-22-09964-s001.zip › ijms-1373377-supplement figures.pdf]

Supplementary figures

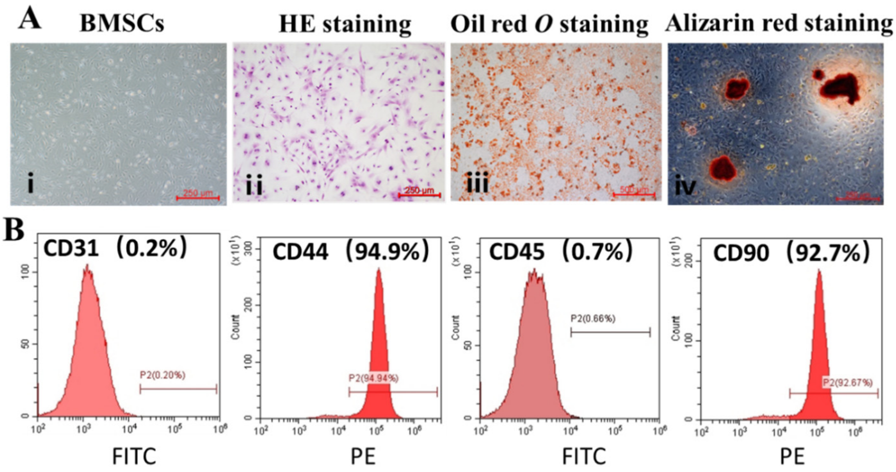

Figure S1. Characteristic of BMSCs. (A) Images of the third generation of primary BMSC (i), HE staining of BMSCs (ii), Oil Red O staining cells (× 40) after 3 weeks culture (iii), and Alizarin red staining cells (× 200) after 4 weeks culture (iv). (B) Flow cytometry assay of BMSCs markers CD44 (94.9%) and CD90 (92.7%), endothelial cell marker CD31 (0.2%), and haematopoietic cell marker CD45 (0.7%).

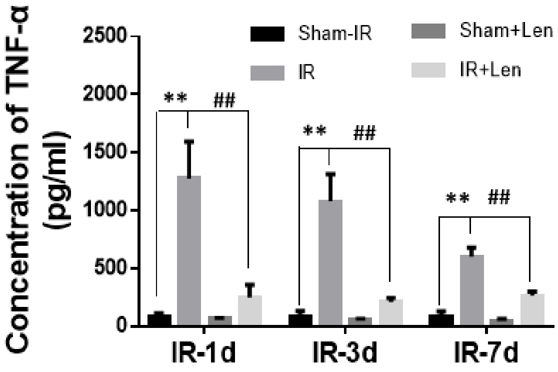

Figure S2. The concentration of TNF-α in mice serum under indicated conditions. ** *P*<0.01 indicated a significant difference with sham group, ## P<0.01 indicated a significant difference with the IR group.
